# PPP2R2D suppresses IL-2 production and Treg function

**DOI:** 10.1172/jci.insight.138215

**Published:** 2020-10-02

**Authors:** Wenliang Pan, Amir Sharabi, Andrew Ferretti, Yinfeng Zhang, Catalina Burbano, Nobuya Yoshida, Maria G. Tsokos, George C. Tsokos

**Affiliations:** 1 Department of Medicine, Beth Israel Deaconess Medical Center (BIDMC) and Harvard Medical School, Boston, Masschusetts, USA.; 2 Department of Pathology, Johns Hopkins University School of Medicine, Baltimore, Maryland, USA.

**Keywords:** Autoimmunity, Immunology, Autoimmune diseases, Phosphoprotein phosphatases, T cells

## Abstract

Protein phosphatase 2A is a ubiquitously expressed serine/threonine phosphatase that comprises a scaffold, a catalytic, and multiple regulatory subunits and has been shown to be important in the expression of autoimmunity. We considered that a distinct subunit may account for the decreased production of IL-2 in people and mice with systemic autoimmunity. We show that the regulatory subunit PPP2R2D is increased in T cells from people with systemic lupus erythematosus and regulates IL-2 production. Mice lacking PPP2R2D only in T cells produce more IL-2 because the *IL-2* gene and genes coding for IL-2–enhancing transcription factors remain open, while the levels of the enhancer phosphorylated CREB are high. Mice with T cell–specific PPP2R2D deficiency display less systemic autoimmunity when exposed to a TLR7 stimulator. While genes related to Treg function do not change in the absence of PPP2R2D, Tregs exhibit high suppressive function in vitro and in vivo. Because the ubiquitous expression of protein phosphatase 2A cannot permit systemic therapeutic manipulation, the identification of regulatory subunits able to control specific T cell functions opens the way for the development of novel, function-specific drugs.

## Abstract 2

PPP2R2D restrains the chromatin opening of IL-2 and its related transcription factors loci to suppress IL-2 production.

## Introduction

Systemic lupus erythematosus (SLE) is an autoimmune disease with a complex pathogenesis. The expression of autoimmunity and organ inflammation involves altered production of cytokines by T cells (1–3). Specifically, decreased IL-2 production may account for the reported decreased cytotoxic T cell function and the decreased function and numbers of Tregs (2), whereas increased production of IFN-γ and IL-17 may contribute directly to organ inflammation (4, 5). Treatment with low doses of IL-2 diminishes renal inflammation and the rate of kidney-infiltrating CD4^+^ T cells in murine lupus nephritis (6). Low-dose IL-2 in the treatment of autoimmune diseases including SLE, rheumatoid arthritis, and multiple sclerosis is in clinical trials (7). IL-2 is a pleiotropic cytokine produced by effector T (T_eff_) cells after antigen activation and is at the crossroads of effector responses, tolerance, and immunotherapy (8). IL-2 transcription is mediated by multiple transcription factors (TFs), including activator protein-1 (AP-1, FOS-JUN family dimers), cAMP responsive element binding protein (CREB), nuclear factor of activated T cells (NFAT) family proteins, NF-κB, ETS-related TF (Elf1), and GA binding protein α chain (GABPA) (9). Tregs have abundant expression of the IL-2 receptor α chain (CD25) but are unable to produce IL-2. IL-2 binds with low affinity to CD25 or to heterodimers of the common γ chain (CD132) and IL-2Rβ (CD122), but receptor affinity increases about 1000-fold when these subunits collectively interact with IL-2 (10). Capture of IL-2 by Tregs is critical for their suppressor function and for limiting the activation of T cells (11).

Protein phosphatase 2A (PP2A) is a ubiquitously expressed and highly conserved serine/threonine phosphatase that is important in multiple cellular processes, including cell division, cytoskeletal dynamics, and various signaling pathways (12). The PP2A core enzyme consists of the scaffold subunit A (PP2A_A_) and the catalytic subunit C (PP2A_C_). To form a functional holoenzyme, the core enzyme interacts with one of the many regulatory subunits (PP2A_B_), which define substrate and tissue specificity (13). The regulatory subunits are classified into 4 major families, identified as B (e.g., PPP2R2A, PPP2R2B, PPP2R2C, and PPP2R2D), B’ (PPP2R5A, PPP2R5B, PPP2R5C, PPP2R5D, and PPP2R5E), B” (PPP2R3A, PPP2R3B, and PPP2R3C), and B’’’ (striatin/PR93) (13). We have previously shown that PP2A mRNA, protein, and catalytic activity are increased in T cells from patients with SLE and that PP2A_C_ is responsible for the dephosphorylation of transcriptional enhancer CREB, which contributes to decreased IL-2 production (14, 15). Although silencing PP2A_C_ or inhibition of its activity increases IL-2 production in T cells (16), in subsequent studies, we found that PP2A_C_ is requisite for Treg function (17) and enables IL-2 signaling during Treg development (18). These apparently contradictory effects make PP2A_C_ inhibitors unlikely to be used for treatment of patients with SLE. However, we previously showed that the PP2A regulatory subunit Bβ (PPP2R2B) is decreased in SLE T cells and accounts for the decreased IL-2 deprivation–induced T cell apoptosis (19). Since the specificity of PP2A function is governed by its use of a particular regulatory subunit (13), we considered that one of the regulatory subunits controls the production of IL-2 in T cells.

We have identified PPP2R2D, a regulatory subunit of PP2A, to negatively regulate IL-2 production in conventional T (T_conv_) cells by controlling chromatin opening of the *IL-2* gene and of genes of multiple TFs that favor IL-2 transcription. PPP2R2D is increased in T cells from patients with SLE, and mice lacking this subunit in T cells develop less autoimmunity. The linkage of PP2A regulatory subunits to specific immune cell functions should enable the development of drugs with higher clinical efficacy.

## Results

### PPP2R2D expression is increased in human T cells following TCR stimulation and suppresses IL-2 production.

**Figure 1:**
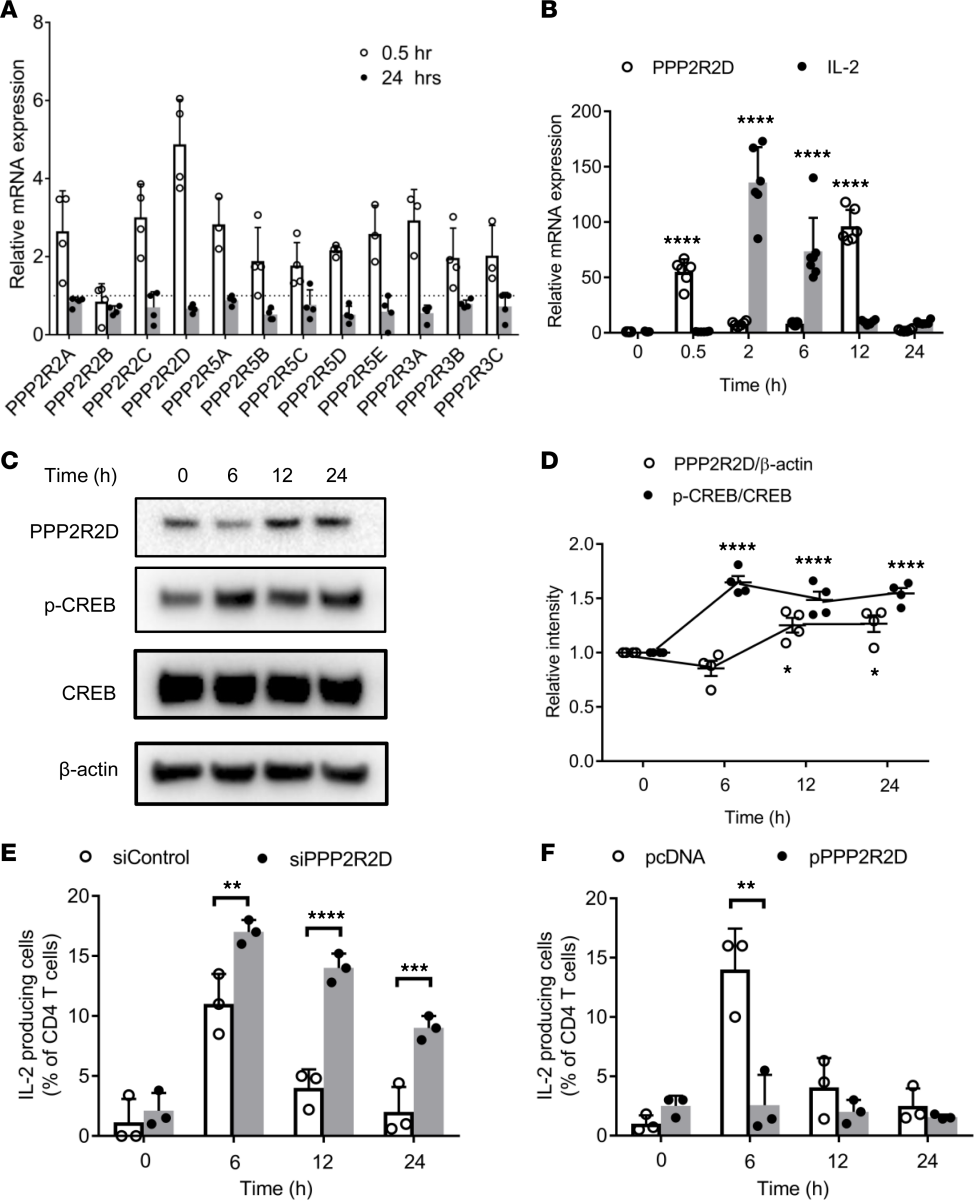
PPP2R2D expression increases and negatively regulates IL-2 production in human T cells following stimulation. Human T cells derived from PBMCs of healthy subjects were stimulated with CD3 (OKT3) and CD28 antibodies. (**A**) The mRNA expression of the different regulatory subunits of PP2A in T cells at 0.5 and 24 hours after stimulation (*n* = 4). Dashed line represents the expression level of each subunit in T cells without stimulation. (**B**) The mRNA expression of *PPP2R2D* and *IL-2* in T cells at 0, 0.5, 2, 6, 12, and 24 hours after stimulation (*n* = 6–7). All the expression levels were normalized to the samples without stimulation. (**C**) Western blot analysis of protein expression levels of PPP2R2D, p-CREB, CREB, and β-actin in T cells at 0, 6, 12, and 24 hours after stimulation. (**D**) Cumulative data (*n* = 4) for quantification of the levels of PPP2R2D and p-CREB in the blots shown in **C**. (**E** and **F**) Intracellular staining of IL-2 production in T cells at 0, 6, 12, and 24 hours after stimulation was analyzed by FACS. Cells were subjected to silencing of PPP2R2D (**E**) or to transfecting with PPP2R2D plasmid (**F**), and they were rested overnight before stimulation for indicated times. *n* = 3. (**B** and **D**) *P < 0.05, ****P < 0.001, when compared with corresponding 0 hour using 2-way ANOVA with Holm-Šidák multiple-comparisons test. (**E** and **F**) **P < 0.01, ***P < 0.001, ****P < 0.001 using multiple *t* test.

Having previously shown that PPP2R2B promotes IL-2 deprivation–induced T cell apoptosis in human T cells (20), we considered that other subunits may control directly the production of IL-2 by T cells. To this end, we determined mRNA expression of several PP2A regulatory subunits in T cells from healthy donors following T cell stimulation with CD3 and CD28 antibodies. mRNA expression increased within 30 minutes for all tested subunits, except for PPP2R2B, but the levels of PPP2R2D increased the most; within 24 hours, mRNA levels for all tested subunits returned to normal (Figure 1A). We looked closely at the kinetics of expression of PPP2R2D mRNA and compared them with those of IL-2. As shown in Figure 1B, PPP2R2D mRNA expression increased at 30 minutes, decreased subsequently at 2 and 6 hours, increased at 12 hours, and decreased at 24 hours, while IL-2 mRNA increased at 2 hours and gradually decreased at 6, 12, and 24 hours. Because PP2A dephosphorylates CREB, a main transcriptional enhancer of IL-2 production, we determined the protein levels of PPP2R2D over time, along with those of phosphorylated CREB (p-CREB), total CREB, and β-actin in lysates extracted from stimulated T cells at various time points. Protein levels of PPP2R2D decreased at 6 hours and subsequently increased at 12 and 24 hours, while the ratio of p-CREB/total CREB increased at 6 hours and subsequently decreased at 12 and 24 hours (Figure 1, C and D).

In order to establish the observed inverse correlation between IL-2 and PPP2R2D expression, we measured IL-2 production following stimulation of T cells in which the expression of PPP2R2D was either silenced (Figure 1E) or overexpressed (Figure 1F). Within 6 hours after stimulation, IL-2 production increased significantly in CD4^+^ T cells (10-fold higher compared with baseline). When PPP2R2D was silenced, the frequency of IL-2–producing CD4^+^ T cells increased further at 6 hours and decreased slowly at 12 and 24 hours after stimulation (Figure 1E). In contrast, PPP2R2D overexpression prevented the induction of IL-2–producing CD4^+^ T cells in response to CD3/CD28 stimulation at all tested time points (Figure 1F). It is noteworthy that neither silencing nor overexpression of PPP2R2D in T cells significantly affected the frequency of IFN-γ– and IL-4–producing CD4^+^ T cells in response to CD3/CD28 stimulation (Supplemental Figure 1, A and B; supplemental material available online with this article; https://doi.org/10.1172/jci.insight.138215DS1). These experiments reveal an important and specific role of PPP2R2D in the production of IL-2.

### PPP2R2D expression is increased in T cells from patients with SLE.

**Figure 2:**
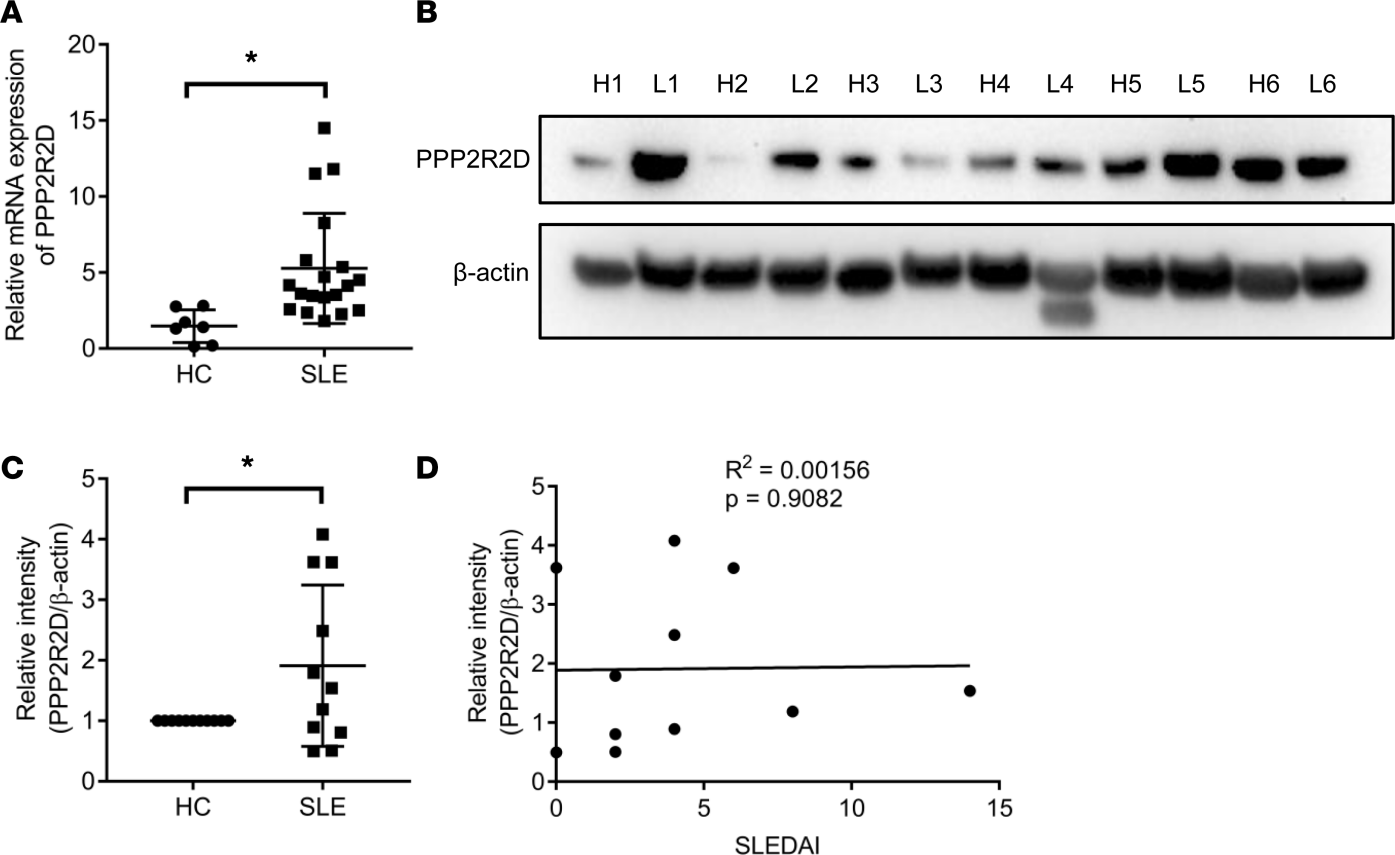
PPP2R2D expression is increased in T cells from patients with SLE. (**A**) The mRNA expression of *PPP2R2D* and in T cells from healthy subjects (*n* = 7) or from patients with SLE (*n* = 19). HC, healthy control. (**B**) Western blot analysis of the protein expression levels of PPP2R2D and β-actin in T cells from patients with SLE or matched healthy donors. Healthy donor 1 (H1) is age-, sex-, and ethnicity-matched with lupus patient 1 (L1); H2 is age-, sex-, and ethnicity-matched with L2, and so on. (**C**) Cumulative data for quantification of the level of PPP2R2D in the blots shown in **B**. Healthy controls (HC), *n* = 11; SLE patients, *n* = 11. (**D**) Pearson correlation analysis showing the relationship between PPP2R2D expression and Systemic Lupus Erythematosus Disease Activity Index (SLEDAI). *P < 0.05 using unpaired *t* test (**A**) or paired *t* test (**C**).

T cells for patients with SLE display high levels of PP2A, which has been linked to decreased IL-2 production. Having shown that PPP2R2D suppressed the production of IL-2, we sought to determine the expression levels of PPP2R2D in SLE T cells. We found that fresh, unstimulated T cells from patients with SLE express high levels of PPP2R2D mRNA compared with T cells from healthy controls (Table 1 and Figure 2A). In parallel to mRNA, the expression of PPP2R2D protein was found increased in T cell lysates from patients with SLE compared with healthy controls (representative Western blots for PPP2R2D and β-actin are shown in Figure 2B, and cumulative data are shown in Figure 2C). However, we did not find a correlation between PPP2R2D expression in SLE T cells and Systemic Lupus Erythematosus Disease Activity Index (SLEDAI) (Figure 2D).

### PPP2R2D deficiency in T cells promotes chromatin remodeling of the IL-2 locus.

To address the function of PPP2R2D in T cells in a definitive manner, we generated C57BL/6J mice in which exon 6 of *PPP2R2D* gene is flanked by loxP (R2D^fl/fl^ mice) using CRISPR/Cas9 technology (Supplemental Figure 2A), as previously described (21). Excision of exon 6 was confirmed by in vitro Cre recombination of PCR products amplified from candidate founders (Supplemental Figure 2B). We then crossed these mice with Lck^Cre^ mice to generate conditional KO mice (Lck^Cre^R2D^fl/fl^) in which PPP2R2D deletion was limited to T cells (Supplemental Figure 2C).

We next determined the role of PPP2R2D in T cell development in R2D^fl/fl^ and Lck^Cre^R2D^fl/fl^ mice. As shown in Supplemental Figure 3, A–C, there were no significant differences in the percentage and absolute numbers of T cell subsets in the thymus from R2D^fl/fl^ and Lck^Cre^R2D^fl/fl^ mice. In order to determine the effect of PPP2R2D deficiency in the survival of thymocytes, we used a CD3 antibody to stimulate thymocytes imitating TCR stimulation during double-positive (DP) cell development. Thymocytes isolated from R2D^fl/fl^ and Lck^Cre^R2D^fl/fl^ mice were stimulated with CD3 antibody overnight and stained for annexin V expression. As shown in Supplemental Figure 3, D–F, CD3 antibody stimulation produced a similar percentage of apoptotic double-negative (DN) or DP cells in R2D^fl/fl^ and Lck^Cre^R2D^fl/fl^ thymocytes. Furthermore, there were no significant differences in the percentage and absolute numbers of T cell subsets in spleens from R2D^fl/fl^ and Lck^Cre^R2D^fl/fl^ mice (Supplemental Figure 3, G–I). These results suggest that PPP2R2D deficiency in T cells does not impair T cell development in the thymus and T cell subset distribution in the spleen.

**Figure 3:**
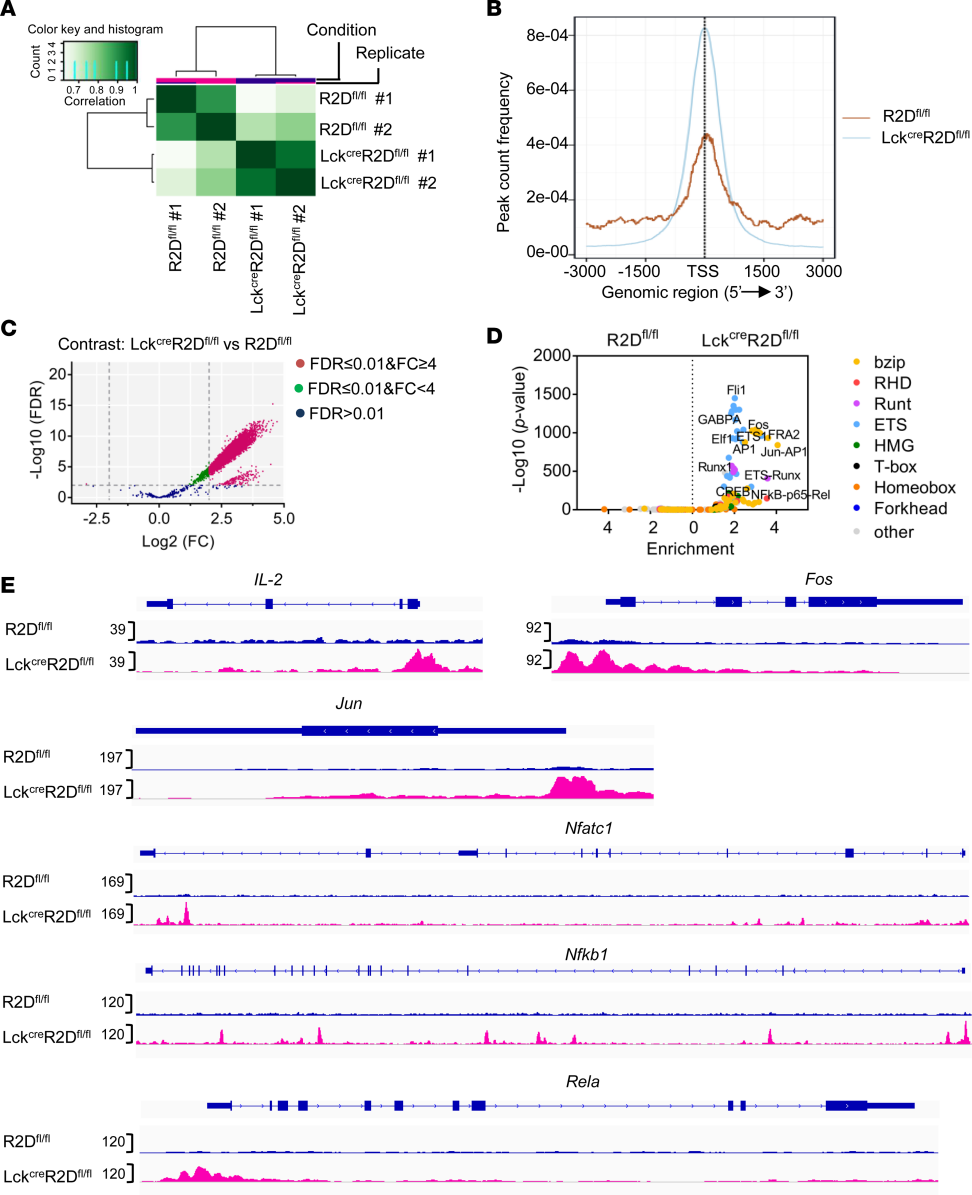
Chromatin accessibility profiles of T_conv_ cells with or without PPP2R2D expression using ATAC-seq analysis. CD4^+^ T_conv_ cells were sorted out from spleens of R2D^fl/fl^ or Lck^Cre^R2D^fl/fl^ mice (*n* = 2 mice/group) and ex vivo stimulated by plate-bound CD3 and CD28 antibodies for 4 hours before being subjected to ATAC-seq. (**A**) Correlation heatmap indicating cross-correlation between each replicate or each group. (**B**) Histogram showing the distance from the nearest transcription start site (TSS) for all ATAC-seq peaks. (**C**) Volcano plot showing differential chromatin accessibility in CD4^+^ T_conv_ cells isolated from R2D^fl/fl^ (WT) and Lck^Cre^R2D^fl/fl^ (KO) mice. Fold change (FC) is calculated as log_2_ (Lck^Cre^R2D^fl/fl^/R2D^fl/fl^). Red indicates sites that were significantly different (adjusted *P* ≤ 0.01, FC ≥ 4). (**D**) Transcription factor (TF) family binding motifs enriched in loci more accessible in Lck^Cre^R2D^fl/fl^ or R2D^fl/fl^ T_conv_ cells; the *x* axis shows the enrichment factor (ratio of the percentage of differential sites with motifs to the percentage of nondifferential sites with motifs), and the *y* axis shows the significance level of enrichment. TF families are indicated by color. (**E**) Accessibility tracks for selected gene loci (*IL-2*, *Fos*, *Jun*, *Nfatc1*, *Nfkb1*, and *Rela*) in R2D^fl/fl^ (up) and Lck^Cre^R2D^fl/fl^ (down) T_conv_ cells were plotted using the integrative genomics viewer (IGV). ATAC-seq data are average of 2 biological replicates at each cell type.

To determine how PPP2R2D regulates T cell signaling at the level of genome-wide transcriptional profile, we performed an assay for transposase-accessible chromatin using sequencing (ATAC-seq) in CD4^+^ T_conv_ cells (Thy1.2^+^CD4^+^CD25^lo^CD127^lo^), which were sorted by flow cytometry from spleens of R2D^fl/fl^ or Lck^Cre^R2D^fl/fl^ mice and stimulated in vitro by plate-bound CD3 and CD28 antibodies for 4 hours. Heatmap clustering of Pearson correlation coefficients revealed a strong correlation between replicates of the same condition in chromatin accessibility but a weaker correlation in profile between R2D^fl/fl^ (WT) and Lck^Cre^R2D^fl/fl^ (PPP2R2D-KO) T_conv_ cells (Figure 3A). We next examined the genomic distribution of ATAC-seq open chromatin peaks. As shown in Figure 3B, a large proportion of ATAC-seq peaks are located close to the transcription start site (TSS), and the number of peaks in Lck^Cre^R2D^fl/fl^ T_conv_ cells was higher than those in R2D^fl/fl^ T_conv_ cells. In order to identify the sites of differential accessibility between R2D^fl/fl^ and Lck^Cre^R2D^fl/fl^ T_conv_ cells, we performed analysis using DESeq2 as previously described (22). Using 2-fold log_2_ difference and FDR less than or equal to 0.01 as cutoffs, we found a larger number of sites differentially open in Lck^Cre^R2D^fl/fl^ T_conv_ as compared with R2D^fl/fl^ T_conv_ cells (Supplemental Table 1 and Figure 3C). To further associate these differentially open sites with the TF networks, we calculated the enrichment of TF-binding motifs at sites that significantly differed in accessibility, using Hypergeometric Optimization of Motif EnRichment (HOMER). At peaks more open in Lck^Cre^R2D^fl/fl^ T_conv_ cells, we found motifs for several members of the ETS (e.g., Fli1, Elf1, GABPA, and ETS1), bzip (e.g., Fos, AP1, FRA2, and CREB1), RHD (e.g., NFAT and NF-κB-p65-Rel), and Runt (e.g., Runx and ETS-Runx) families to be most significantly enriched (Figure 3D). Interestingly, most of these TFs are centered on IL-2, whose transcription is mediated by multiple TFs, including AP-1 (FOS-JUN family dimers), CREB, NFAT family proteins, NF-κB, Elf1, and GABPA (9). Furthermore, sequencing read densities at genes encoding *IL-2* and its related TFs, such as *Jun*, *Fos*, *Nfatc1*, *Nfkb1*, and *Rela*, were increased in Lck^Cre^R2D^fl/fl^ T_conv_ cells in comparison with those in R2D^fl/fl^ T cells (Figure 3E), demonstrating that lack of PPP2R2D expression promoted the chromatin accessibility of IL-2 and its related TFs.

### PPP2R2D deficiency enhances IL-2 production in T cells.

**Figure 4:**
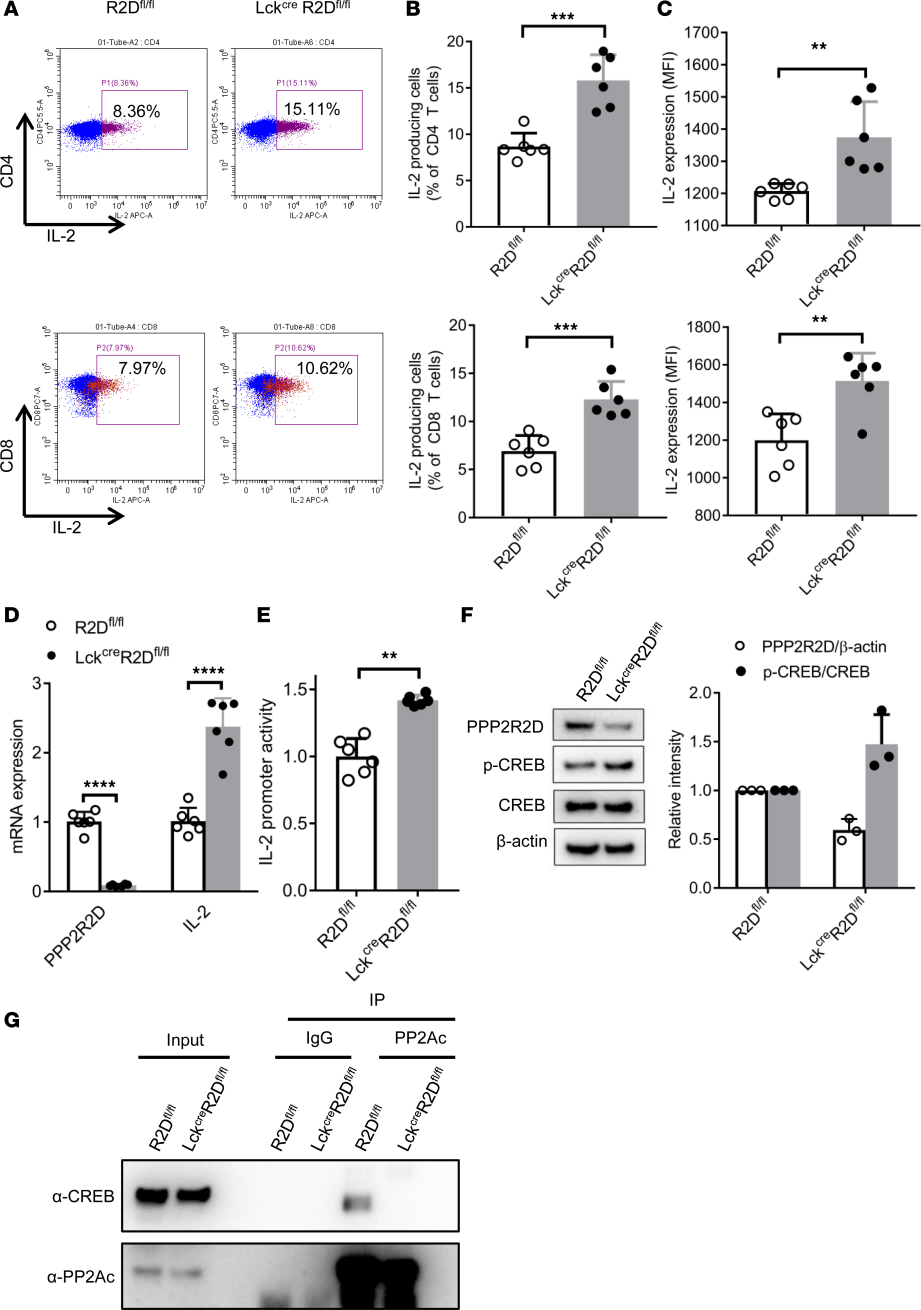
PPP2R2D deficiency enhances IL-2 production by T cells. Splenic CD4^+^ or CD8^+^ T cells were isolated from R2D^fl/fl^ and Lck^Cre^R2D^fl/fl^ mice. (**A**–**C**) Splenic CD4^+^ or CD8^+^ T cells were stimulated with phorbol myristate acetate (PMA)/ionomycin and brefeldin A for 4 hours before subjected to FACS analysis of intracellular staining of IL-2 production. Representative flow cytometry plots were shown in **A**. Cumulative data (*n* = 6 mice/group) from individual mice depicting the percentages of IL-2–producing cells (**B**) and the expression of IL-2 (**C**) were presented. MFI, mean fluorescence intensity. (**D**) The mRNA expression level of *PPP2R2D* and *IL-2* in R2D^fl/fl^ and Lck^Cre^R2D^fl/fl^ T cells. Splenic CD4^+^ T cells were stimulated with plate-bound anti-CD3 and anti-CD28 for 6 hours before extraction of RNA for quantitative PCR analysis. Data are shown as *n* = 3 mice/group with 2 technical replicates for each mouse. (**E**) The promoter activity of IL-2 in R2D^fl/fl^ and Lck^Cre^R2D^fl/fl^ T cells. Splenic CD4^+^ T cells were nucleofected with IL-2 promoter by using Amaxa Nucleofector electroporation, rested overnight, and stimulated with plate-bound anti-CD3 and anti-CD28 for 24 hours before measurement of luciferase activity. Data are shown as *n* = 3 mice/group with 2 technical replicates for each mouse. (**F** and **G**) Splenic CD4^+^ T cells were stimulated with plate-bound anti-CD3 and anti-CD28 for 6 hours. (**F**) Western blot analysis of PPP2R2D, p-CREB, CREB, and β-actin in T cells. Representative immunoblots and cumulative data (*n* = 3 mice/group, right) are presented. (**G**) Co-IP analysis of PP2A_C_ and CREB in T cells. The blot is representative of 3 independent experiments. **P < 0.01, ***P < 0.001, ****P < 0.001 using unpaired *t* test.

In order to confirm the above-mentioned findings, R2D^fl/fl^ and Lck^Cre^R2D^fl/fl^ spleen CD4^+^ or CD8^+^ T cells were stimulated in vitro with phorbol myristate acetate (PMA)/ionomycin, and brefeldin A for 4 hours before staining for intracellular cytokines, followed by FACS analysis. In line with ATAC-seq results, PMA/ionomycin induced a higher percentage of IL-2–producing cells (Figure 4, A and B) and expression levels of IL-2 (Figure 4C) in both Lck^Cre^R2D^fl/fl^ CD4^+^ and CD8^+^ T cells compared with those in R2D^fl/fl^ T cells. However, there was no significant difference in the percentage of IFN-γ–producing cells and the expression level of IFN-γ in both CD4^+^ and CD8^+^ T cells from Lck^Cre^R2D^fl/fl^ and R2D^fl/fl^ mice following PMA/ionomycin stimulation (Supplemental Figure 4, A–C), which is in agreement with our findings in human T cells. In addition, lack of PPP2R2D expression did not affect the in vitro differentiation of CD4^+^ naive T cells into either Th cells Th1 and Th17 or Tregs (Supplemental Figure 4, D–F). Next, we investigated the transcription of IL-2 and found that, following stimulation with CD3 and CD28 antibodies, Lck^Cre^R2D^fl/fl^ CD4^+^ T cells displayed higher IL-2 mRNA levels (Figure 4D) and promoter activity (Figure 4E) compared with R2D^fl/fl^ CD4^+^ T cells. CREB is an important regulator of IL-2 transcription. p-CREB at Ser133 residue enhances the activity of the IL-2 promoter through direct binding to the –180 site of the IL-2 promoter (23) or by upregulating the expression of other IL-2 TFs cJUN, cFOS, FRA2, and FOSB (9). Our previous data show that PP2A is able to dephosphorylate CREB (16); thus, we interrogated whether PPP2R2D, the regulatory subunit of PP2A, is involved in this process. Notably, Western blot analysis showed that PPP2R2D-deficient (Lck^Cre^R2D^fl/fl^) CD4^+^ T cells displayed increased p-CREB levels (Figure 4F) in comparison with WT (R2D^fl/fl^) CD4^+^ T cells. This is probably caused by the decreased binding of PP2A_C_ to CREB in the absence of the regulatory subunit PPP2R2D (Figure 4G). These results suggest that PPP2R2D deficiency is involved in the maintenance of p-CREB, which — along with the aforementioned opening of a number of IL-2 transcriptional enhancers — augments IL-2 production.

### PPP2R2D deficiency in T cells alleviates imiquimod-induced lupus-like pathology in mice.

**Figure 5:**
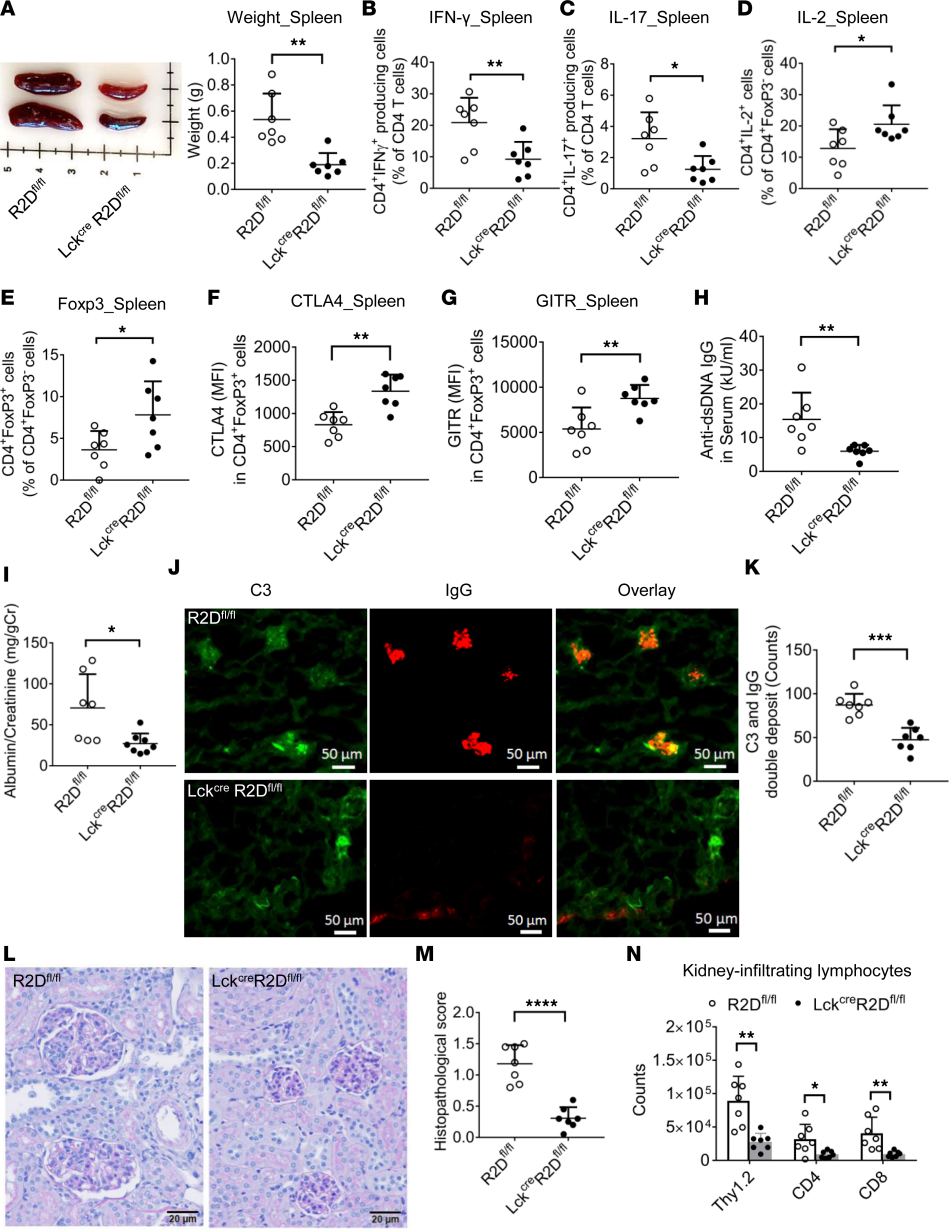
PPP2R2D deficiency in T cells mitigates imiquimod-induced lupus-like pathology in mice. Topical imiquimod was applied to the ear skin of R2D^fl/fl^ and Lck^Cre^R2D^fl/fl^ mice (*n* = 7/group) for 8 weeks. (**A**) The representative picture and cumulative data of the weight of spleens. FACS analysis of the percentage of CD3^+^CD4^+^IFN-γ^+^ (**B**), CD3^+^CD4^+^IL-17A^+^ (**C**)**,** CD3^+^CD4^+^IL-2^+^ (**D**), and CD3^+^CD4^+^FoxP3^+^ (**E**) cells in spleens. (**F** and **G**) The expression levels of Treg markers CTLA-4 (**F**) and GITR (**G**) in splenic Tregs (CD3^+^CD4^+^FoxP3^+^) were determined by FACS. (**H** and **I**) The anti-dsDNA IgG level in serum (**H**) and the levels of albumin and creatinine in urine (**I**) were measured by ELISA. (**J** and **K**) The deposition of complement 3 (C3) and IgG in glomeruli was determined by immunofluorescence staining. Representative figures (**J**) and cumulative data (**K**) depicting numbers of glomeruli with C3 and IgG double deposition in coronal sections of kidney. Scale bar: 50 μm. (**L**) Representative H&E staining of kidney tissues. Scale bar: 20 μm. (**M**) Cumulative data elucidating the histopathologic scores for glomerulonephritis. (**N**) Kidney-infiltrating lymphocytes including total T cells (Thy1.2), CD4^+^ T cells, and CD8^+^ T cells were counted and analyzed by FACS. *P < 0.05, **P < 0.01, ***P < 0.001, ****P < 0.001 using unpaired *t* test.

IL-2 production is deficient in the T cells of patients with SLE (2), and treatment with low doses of IL-2 diminishes disease activity by promoting Treg function (7). To determine the role of PPP2R2D-deficient T cells in regulation of lupus-related pathology, we applied imiquimod, a TLR7 stimulator, to the ear skin of 8-week-old R2D^fl/fl^ and Lck^Cre^R2D^fl/fl^ mice to induce lupus-like disease, as previously described (24). As shown in Figure 5A, imiquimod-treated Lck^Cre^R2D^fl/fl^ mice showed significantly reduced size and weight of spleens as compared with imiquimod-treated R2D^fl/fl^ mice. The percentages of IFN-γ–producing (CD3^+^CD4^+^IFN-γ^+^ and CD3^+^CD8^+^IFN-γ^+^; Figure 5B and Supplemental Figure 5A) and IL-17A–producing (CD3^+^CD4^+^IL-17A^+^ and CD3^+^CD8^+^IL-17A^+^; Figure 5C and Supplemental Figure 5B) cells were significantly decreased in the imiquimod-treated Lck^Cre^R2D^fl/fl^ mice as compared with imiquimod-treated R2D^fl/fl^ mice. In contrast, the percentages of IL-2–producing cells (CD3^+^CD4^+^IL-2^+^; Figure 5D) and of the CD3^+^CD4^+^FoxP3^+^ cells (Tregs, Figure 5E) were significantly increased in the imiquimod-treated Lck^Cre^R2D^fl/fl^ mice in comparison with imiquimod-treated R2D^fl/fl^ mice. Interestingly, the expression levels of Treg markers CD25, CTLA-4, and GITR were increased in imiquimod-treated Lck^Cre^R2D^fl/fl^ Tregs compared with the imiquimod-treated R2D^fl/fl^ Tregs (Supplemental Figure 5C and Figure 5, F and G). Moreover, imiquimod-treated Lck^Cre^R2D^fl/fl^ mice developed less lupus-like nephritis, as evidenced by the decreased anti–dsDNA IgG levels in the serum (Figure 5H), proteinuria (Figure 5I), deposition of complement C3 and IgG in glomeruli (Figure 5, J and K; Supplemental Figure 5D), histologic signs of glomerulonephritis (Figure 5, L and M), and number of kidney-infiltrating lymphocytes (Figure 5N) when compared with those in the imiquimod-treated R2D^fl/fl^ mice. Collectively, PPP2R2D deficiency in T cells protects against lupus-like disease in mice.

### PPP2R2D deficiency in T cells potentiates the function of Tregs.

**Figure 6:**
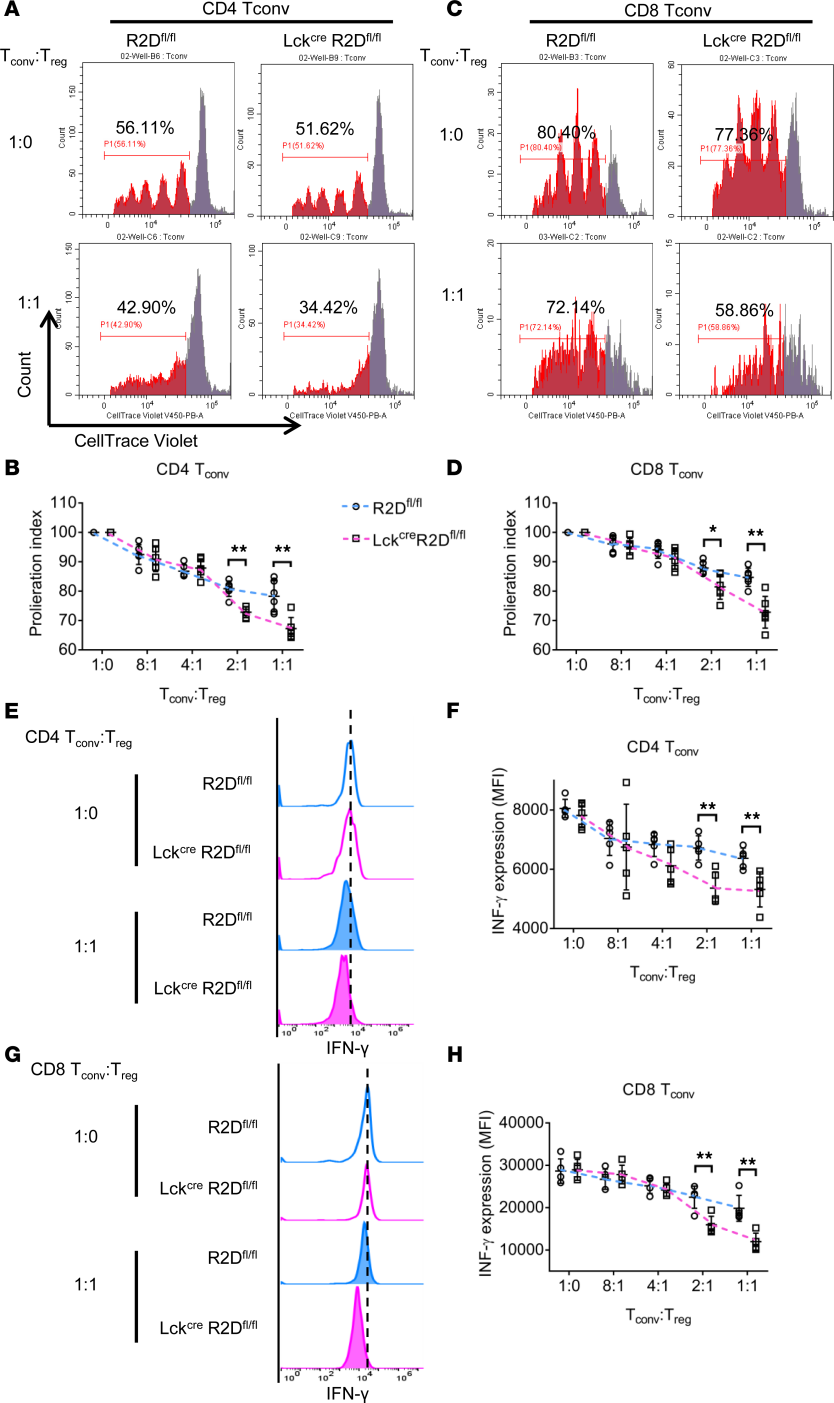
Loss of PPP2R2D expression in T cells enhances the suppressive capacity of Tregs. In vitro suppression of R2D^fl/fl^ or Lck^Cre^R2D^fl/fl^ CD4^+^ T_conv_ or CD8^+^ T_conv_ cells by R2D^fl/fl^ or Lck^Cre^R2D^fl/fl^ Tregs after incubation together at various ratios. (**A**–**D**) Proliferation index of CD4^+^ T_conv_ (**A** and **B**) or CD8^+^ T_conv_ (**C** and **D**) cells is shown. Representative flow charts of the dilution of fluorescent dye CellTrace Violet (**A** and **C**) and cumulative data (**B** and **D**) are shown. *n* = 6 mice/group. (**E**–**H**) Expression of IFN-γ in CD4^+^ T_conv_ (**E** and **F**) or CD8^+^ T_conv_ (**G** and **H**) cells is shown. Representative flow charts of the expression of IFN-γ (**E** and **G**) and cumulative data (**F** and **H**) are shown. *n* = 5 mice/group. *P < 0.05, **P < 0.01 using multiple *t* test.

Our aforementioned findings that the percentage of Tregs in the spleens (imiquimod-induced lupus-like disease model, Figure 5E) was increased in Lck^Cre^R2D^fl/fl^ as compared with R2D^fl/fl^ mice raised the question of whether lack of PPP2R2D expression in Tregs affects the expression of genes responsible directly for their function. To test this possibility, we performed ATAC-seq analysis of Tregs (Thy1.2^+^CD4^+^CD25^hi^CD127^lo^) that were isolated from spleens of R2D^fl/fl^ or Lck^Cre^R2D^fl/fl^ mice by flow cytometry and stimulated in vitro with IL-2 and plate-bound CD3 and CD28 antibodies for 4 hours. As shown in Supplemental Figure 6A, a large proportion of ATAC-seq peaks are located close to the TSS, and the number of peaks in Lck^Cre^R2D^fl/fl^ T cells is similar to those in R2D^fl/fl^ T cells. Using a *P* value less than or equal to 0.05 as a cutoff, differential accessibility analysis revealed limited sites (genes) differentially open between Lck^Cre^R2D^fl/fl^ and R2D^fl/fl^ Tregs (Supplemental Figure 6B). None of these genes (*Tcstv3*, *Mir101c*, *Cldn14*, *Lrrc4c*, *Gm5458*, *Vmn2r123*, *Lrrc4c*, *Cr2*, and *Nkx2-6*) are known to be involved in the suppressive function of Tregs, indicating that PPP2R2D deficiency in Tregs does not affect the expression of genes known to account for their suppressive function. These findings demonstrate that PPP2R2D controls the expression of genes related to the expression of IL-2 and the *Il-2* gene itself without affecting the expression of genes known to define Treg function. Instead, we considered that the increased levels of IL-2 potentiate the suppressive function of Tregs. Indeed, PPP2R2D-deficient (Lck^Cre^R2D^fl/fl^) Tregs displayed a more suppressive effect on the proliferation of PPP2R2D-deficient CD4^+^ or CD8^+^ T_conv_ cells (Figure 6, A–D) and the expression of IFN-γ by these cells (Figure 6, E–H) when compared with the suppressive effect of WT (R2D^fl/fl^) Tregs.

## Discussion

We present evidence that PPP2R2D, a regulatory subunit of PP2A, negatively regulates IL-2 production in T_conv_ cells by controlling the chromatin opening of *IL-2* and related multiple TFs, which enhance *IL-2* transcription. Mechanistically, we demonstrate that PPP2R2D deficiency in T cells potentiates the suppressive function of Tregs. At the translational level, we show that PPP2R2D deficiency in T cells alleviates imiquimod-induced lupus-like pathology.

SLE is an autoimmune disorder of unknown etiology characterized by diverse T_eff_ cell dysfunction involving both CD4^+^ and CD8^+^ T cells (1–3). Specifically, decreased IL-2 production accounts for the reported decreased cytotoxic T cell function and the decreased function and numbers of Tregs (2), whereas increased production of IFN-γ and IL-17 may contribute directly to organ inflammation (4, 5). We have previously established that the catalytic subunit PP2A_C_ of PP2A is increased in T cells isolated from patients with SLE and causes decreased production of IL-2 by dephosphorylating the transcriptional enhancer p-CREB (16). In subsequent studies, we showed that transgenic mice overexpressing PP2A_C_ in T cells develop glomerulonephritis (25), which was attributed to epigenetic modification of proinflammatory genes (26).

The abundance of PP2A in all cells and the large number of its regulatory subunits (more than 20) point to a complicated landscape whereby PP2A controls cell function (13). Previously, we showed that PPP2R2B is decreased in SLE T cells and accounts for decreased IL-2 deprivation–induced T cell apoptosis (19). In this report, we identify that PPP2R2D is overexpressed in T cells from patients with SLE and functions as a negative regulator of IL-2 production in T cells. In addition, under TCR stimulation, PPP2R2D is mostly upregulated, whereas PPP2R2B is not affected. Therefore, in T cells, 2 subunits (PPP2R2B and PPP2R2D) control distinct functions. With regard to the study of T cells from patients with SLE, the involvement of PP2A in the immunopathogenesis of the disease appears to be even more complicated in view of the fact that the expression of PPP2R2B is decreased (20), whereas the expression of PPP2R2D is increased, as shown in this study.

In this report, we provide evidence that PPP2R2D, in different T cell subsets (T_conv_ cells and Tregs), plays differential essential roles. Upon ATAC-seq, we found that lack of PPP2R2D expression in T_conv_ cells boosts the chromatin opening of a large number of genes, while in Tregs, chromatin remodeling is limited. These findings demonstrate that PPP2R2D is more important in the direct regulation of T_conv_ cell function (IL-2 production) rather than Treg function. Although silencing PP2A_C_ or inhibition of its activity increased IL-2 production in T cells (16), in subsequent studies, we found that PP2A_C_ is requisite for Treg function (17) and enables IL-2 signaling during Treg development (18). These apparently contradictory data make PP2A_C_ inhibitors unlikely to be used for treatment of patients with SLE. Data presented in this study demonstrate that PPP2R2D deficiency enhances the production of IL-2 in T_conv_ cells without directly affecting Tregs, thus making PPP2R2D a promising target to treat IL-2–deficient autoimmune and inflammatory disorders. In fact, we present evidence that PPP2R2D deficiency in T cells alleviates imiquimod-induced lupus-like pathology in mice. Our findings are in conceptual agreement with a recent report demonstrating that T cells with silenced PPP2R2D produced more IL-2 and other cytokines when transferred into mice along with melanoma cells (27).

IL-2 is produced after antigen activation and is involved in effector responses and immune tolerance (8). On one hand, IL-2 is needed for the expansion of T_eff_ cells (28, 29), and on the other hand, IL-2 drives the development of CD4^+^FOXP3^+^ Tregs, which have suppressor function and mediate immune tolerance (30, 31). IL-2 consumption by Tregs has been suggested to be essential for the suppressor function of Tregs by causing the death of activated T cells (11, 32). Our data from the T_conv_ and Tregs coculture experiments show that PPP2R2D deficiency in T cells potentiates the suppressive function of Tregs, and this is probably due to the fact that more IL-2 is produced by PPP2R2D-deficient T cells. Low-dose IL-2 treatment has been shown to expand the Treg population in patients with SLE (33, 34) and is used in clinical trials for treatment of autoimmune diseases, including SLE, rheumatoid arthritis, and multiple sclerosis (7).

In summary, we have shown that PPP2R2D, a regulatory subunit of PP2A, suppresses the production of IL-2 and Treg activity, and its specific targeting should increase IL-2 production and Treg activity in autoimmune diseases. Since PPP2R2D is increased in people with SLE, its targeted inhibition should have therapeutic value, at least in the subgroup of patients with elevated expression of this subunit. The fact that PP2A is present in every cell and is involved in numerous cell functions argues for the need to link regulatory subunits to specific cell functions. Patients with autoimmune diseases who have increased levels of PPP2R2D in T cells could benefit from proper inhibitors.

## Methods

### Human subjects.

Patients who fulfilled the criteria for the diagnosis of SLE by the American College of Rheumatology were enrolled in this study. Age-, sex-, and ethnicity-matched healthy donors were chosen as controls.

### Human T cell isolation.

The blood from study subjects was incubated for 30 minutes with a RosetteSep Human T Cell Enrichment Cocktail (STEMCELL Technologies) that contained a tetrameric Ab mixture against CD14, CD16, CD19, CD56, and glyA that attaches non–T cells to erythrocytes. Lymphocyte separation medium (17-829E, Lonza) was subsequently used to separate these complexes from T cells. Cells were then either directly lysed to extract protein and RNA or cultured for following study.

### Human T cell culture and stimulation.

T cells (1 × 10^6^ to 2.5 × 10^6^) were cultured in RPMI 1640 medium, (10-040-CVR, Corning), 10% heat-inactivated FBS (SH3007003HI, Hyclone), 2 mM glutamine (25-030-081, Gibco, Thermo Fisher Scientific), 100 U/mL penicillin, and 100 μg/mL streptomycin (15140122, Thermo Fisher Scientific) at 37°C, 5% CO_2_. T cells were stimulated with plate-bound OKT3/anti-CD3 (5 μg/mL, 317325, BioLegend) and anti-CD28 (1 μg/mL, CD28.2, BioLegend) for the indicated time.

### Electroporation in T cells.

Control siRNA (catalog 4404021) and siRNA targeting PPP2R2D (catalog AM16708) were purchased from Ambion Inc. PPP2R2D plasmid was obtained from Addgene (plasmid 13804). Plasmid or siRNA electroporation in primary human T cells were carried out using the Nucleofector system (Lonza). Five million freshly isolated T cells were resuspended in 100 μL of Nucleofector solution, and the respective amounts of plasmid or siRNA were added. Cells were transfected using the program U-014 and were rescued immediately in prewarmed RPMI medium (10-040-CVR, Corning) supplemented with 10% FBS (SH3007003HI, Hyclone) and 1% penicillin/streptomycin (15140122, Thermo Fisher Scientific). Cells were then stimulated using anti-CD3 (317325, BioLegend) and anti-CD28 (CD28.2, BioLegend) for indicated times and stained for FACS analysis.

### Quantitative PCR.

Total RNA was isolated from purified T cells with RNeasy Plus Micro Kit (QIAGEN). The isolated RNA was transcribed into cDNA using the using the RNA to cDNA premix (Clontech) according to the manufacturer’s instructions. SYBR green was purchased from Roche, and the assays were performed on 96-well reaction plates (Invitrogen). The real-time PCR was performed on StepOnePlus system (Invitrogen). In all experiments, β-actin was used as reference gene to normalize gene expression. Primers are shown in Supplemental Table 2.

### Flow cytometry.

Cells were stained with fluorescence-tagged antibodies purchased from eBioscience, BD Biosciences, Tonbo Bioscience, or BioLegend (Supplemental Table 3) and analyzed using Cytoflex flow cytometer. Flow cytometry data were analyzed using CytExpert version 2.0. For intracellular cytokine staining, cells were stimulated with 50 ng/mL of PMA, 1 μM of ionomycin, and 1 μg/mL of brefeldin A for 4 hours in the presence of brefeldin A; they were then harvested, fixed, and stained with BD Cytofix/Cytoperm Fixation/Permeabilization Solution Kit.

### Mice.

PPP2R2D flox (R2D^fl/fl^) mice were generated using CRISPR/Cas9 technology, as previously described (21). Briefly, pronuclear zygotes were microinjected with Cas9 protein, 2 gRNAs targeting intronic regions flanking exon 6 of PPP2R2D, and donor single-stranded DNA oligonucleotides each containing a loxp consensus sequence and restriction enzyme sites. Litters were crossed with C57BL/6J mice (The Jackson Laboratory) and screened using PCR, followed by restriction enzyme digestion to identify candidate founders (R2D^fl/fl^). Excision of exon 6 was confirmed by in vitro Cre recombination of PCR products amplified from candidate founders. To generate PPP2R2D conditional KO mice, R2D^fl/fl^ mice were crossed with Lck^Cre^ (distal promoter) mice to have Lck^Cre^R2D^fl/fl^ mice, where PPP2R2D is deleted in T cells. Both age- and sex-matched male and female mice at the age of 8–12 weeks (unless indicated otherwise in the figure legend) were used for experiments. All mice were bred and housed in a specific pathogen–free (SPF) environment in a barrier facility, in accordance with the BIDMC IACUC.

### Induction of lupus-like disease.

R2D^fl/fl^ or Lck^Cre^R2D^fl/fl^ mice received a topical treatment of 1.25 mg of 5% imiquimod cream (Perrigo), a TLR7 stimulator, on the skin of the right ear 3 times a week, from 8 to 16 weeks of age, which induces lupus-like disease, as previously described (24). At the end of the experiment, urine, serum, spleen, and kidney were collected for indicated analyses.

### ELISAs.

The amounts of albumin and creatinine in urine, as well as serum IgG autoantibody against dsDNA, were determined using standardized ELISAs. The following ELISA kits were used: mouse anti–dsDNA IgG ELISA (Alpha Diagnostic International Inc.), Parameter Creatinine Kit (R&D Systems), and Mouse Albumin ELISA Quantitation Set (Bethyl Laboratories). All procedures were performed according to the manufacturer’s instructions.

### Single cell isolation.

Spleens were excised and weighed, and single-cell suspensions were obtained. Kidneys were perfused with PBS and digested with collagenase type IV (300 U/mL, Worthington Biochemical) and DNAse I (100 μg/mL, Roche) in HBSS for 30 minutes at 37°C.

### Renal histopathology.

Histopathologic assessment was performed as previously described (35). Twenty glomeruli per kidney section were evaluated, and the average score was calculated in each mouse. The changes of each glomerulus were scored semiquantitatively on a scale of 0–3: 0, normal (30–40 cells per glomerulus); 1, mild (41–50 cells/glomerulus and/or minor exudates); 2, moderate (51–60 cells per glomerulus, hyalinosis, and/or moderate exudates); and 3, severe (>60 cells per glomerulus, segmental or global sclerosis, necrosis, crescent formation, and/or heavy exudates).

### Complement C3 and IgG staining.

Coronal sections of frozen kidneys (6 μm) were fixed, stained with Fluorescein-conjugated goat IgG to mouse complement C3 (SKU 0855500, MP Biomedicals; 1:100) and Texas Red goat anti–mouse IgG antibody (T-6390, Invitrogen; 1:100), and mounted with VECTASHIELD Antifade Mounting Medium with DAPI (Vector Laboratories, Maravai LifeSciences). Then, the images of an entire coronal section were captured with an All-in-One Fluorescence Microscope (BZ-X800E, Keyence) and analyzed using BZ-X800 analyzer. The number of glomeruli with C3 and IgG double deposition in the coronal section of kidney were counted.

### Apoptosis detection.

Two million thymocytes were cultured in 96-well plates with 1 μg/mL of anti-CD3 (145-2C11; BioLegend) overnight. Then, cells were stained with surface antigens (CD4, CD8) at 4°C for 15 minutes, washed with cold PBS, and stained with 2 μL of annexin V (BD Pharmingen, 556570) in 200 μL of 1× binding buffer at room temperature for 15 minutes before being collected for FACS analysis. The FACS data were analyzed by gating single cells and exclusion of dead cells.

### Mouse T cell isolation and culture.

Mouse CD4^+^ or CD8^+^ T cells were isolated using EasySep Mouse CD4^+^ (catalog 19852) or CD8^+^ (catalog 19853) T Cell Isolation Kit (STEMCELL Technologies), per manufacturer’s instruction. Cells were cultured in RPMI 1640 medium, 10% heat-inactivated FBS, 2 mM glutamine, 100 U/mL penicillin, 100 μg/mL streptomycin, and 2-mercaptoethanol (50 μM) at 37°C, 5% CO_2_.

### In vitro T cell differentiation.

Naive CD4^+^ T cells were purified by mouse CD4^+^CD62L^+^ T Cell Isolation Kit II (Miltenyi Biotec). Purified naive T cells were stimulated with plate-bound goat anti–hamster IgG (SKU 0856984, MP Biomedicals), soluble anti-CD3 (0.25 μg/mL,145-2C11; BioLegend), and anti-CD28 (0.5 μg/mL, 37.51; BioLegend) for Th0-nonpolarized condition culture. In addition to Th0-nonpolarized condition, the following stimulation was used for each polarized condition: IL-12 (20 ng/mL; R&D Systems) and anti–IL-4 (10 μg/mL, C17.8; BioLegend) for Th1; IL-6 (3 ng/mL; R&D Systems), TGF-β1 (0.3 ng/mL; R&D Systems), anti–IL-4 (10 μg/mL, C17.8; BioLegend), and anti–IFN-γ (10 μg/mL; XMG1.2; BioLegend) for Th17; and IL-2 (20 ng/mL; R&D Systems), TGF-β1 (3 ng/mL), anti–IL-4 (10 μg/mL), and anti–IFN-γ (10 μg/mL) for Treg.

### In vitro suppression assay.

T_conv_ cells (Thy1.2^+^CD4^+^CD25^lo^CD127^lo^ and Thy1.2^+^CD8^+^CD25^lo^CD127^lo^) and Tregs (Thy1.2^+^CD4^+^CD25^hi^CD127^lo^) were sorted from freshly isolated mouse splenocytes by FACSAria II and stained with CellTrace Violet and CFSE, respectively. A total of 4 × 10^4^ T_conv_ cells was cultured with graded numbers of Tregs in the presence of 1 × 10^5^ irradiated, T cell–depleted splenocytes, and 1 μg/mL anti-CD3 (145-2C11; BioLegend) and anti-CD28 (37.51; BioLegend) in a 96-well, round-bottom plate for 72 hours. Cell proliferation of T_conv_ cells was determined by flow cytometry based on the dilution of fluorescence intensity of CellTrace Violet of the gated cells.

### Luciferase reporter assay.

Mouse IL-2 promoter region (747 bp) was cloned into pGL3 Basic vector by GenScript. R2D^fl/fl^ or Lck^Cre^R2D^fl/fl^ CD4^+^ T cells were nucleofected with IL-2 promoter by using Amaxa Nucleofector electroporation, rested overnight, and stimulated with plate-bound anti-CD3 (1 μg/mL) and anti-CD28 (1 μg/mL) for 24 hours. Then, cells were harvested and lysed, and luciferase activity was detected by GloMax-Multi Detection System (Promega) using the Dual-Luciferase Reporter Assay Kit (E1910, Promega). Luciferase activity was normalized to renilla luciferase activity, as well as to the R2D^fl/fl^ CD4^+^ T cells.

### Western blotting.

Cell lysates were prepared using RIPA buffer (Boston BioProducts) containing protease cocktail inhibitor (cOmplete Mini EDTA-free, Roche) and phosphatase cocktail inhibitor (PhosSTOP, Roche). Protein concentration was determined by coomassie protein assay reagent (MilliporeSigma). Total protein (20 μg) was resolved by a NuPAGE 4%–12% Bis-Tris gel (Invitrogen), and transferred to PVDF membrane (Thermo Fisher Scientific). After blocking with 5% nonfat milk (M-0841, LabScientific), the membrane was incubated with primary antibody overnight at 4°C. Subsequently, the membrane was incubated with secondary antibody for 90 minutes at room temperature. Western ECL substrate (1705061, Bio-Rad) was used to develop the immunoblot. The picture was captured and analyzed by Image Lab (Version 5.2.1) using ChemiDoc XRS+ System (Bio-Rad). The results were quantified by plotting the intensity of the band. β-Actin was used as the loading control. Primary antibodies against CREB (catalog 9197) and PP2A_C_ (catalog 2259) were purchased from Cell Signaling Technology, p-CREB (catalog 06-519) and β-actin (catalog A5316) were from MilliporeSigma, and PPP2R2D (catalog ab181071) was from Abcam.

### Co-IP.

Co-IP experiments were conducted using Pierce Classic Magnetic IP/Co-IP Kit (88804, Thermo Fisher Scientific) according to manufacturer’s instructions. Briefly, protein lysates were prepared using ice-cold IP lysis buffer containing protease cocktail inhibitor (cOmplete Mini EDTA-free, Roche). Then, the antigen/antibody mixture was made by overnight incubation of protein lysate with an antibody either against PP2A_C_ (05-421, MilliporeSigma) or normal mouse IgG (sc-2025, Santa Cruz Biotechnology Inc.) as indicated. Subsequently, the mixture was added to the tube containing prewashed magnetic beads and incubated for 1 hour at room temperature under continuous mixing. The beads were then collected on a magnetic stand and washed, and the protein complex was eluted from the beads for Western blot analysis.

### ATAC-seq.

ATAC-seq was done as described by Corces et al. (36). Briefly, R2D^fl/fl^ and Lck^Cre^R2D^fl/fl^ CD4^+^ T_conv_ cells (Thy1.2^+^CD4^+^CD25^lo^CD127^lo^), which were sorted out by flow cytometry, were stimulated with plate-bound CD3 (1 μg/mL) and CD28 (1 μg/mL) antibodies for 4 hours. Tregs (Thy1.2^+^CD4^+^CD25^hi^CD127^lo^), which were sorted out by flow cytometry, were stimulated with IL-2 (20 ng/mL) and with plate-bound CD3 (1 μg/mL) and CD28 (1 μg/mL) antibodies for 4 hours. We followed the Omni-ATAC protocol (36), and deep sequencing was performed using PE35 bp reads on Illumina NextSeq500 at Boston Nutrition Obesity Research Center Functional Genomics Core.

ATAC-seq data were evaluated for quality using FASTQC. Adapter sequences were trimmed from the raw reads with CutAdapt (v2.7). Trimmed reads of each sample were mapped to the reference house mouse genome build GRCm38 (mm10) by using Bowtie2 (v2.3.5). BAM files were generated and sorted by query name with SAMtools (v1.9). Genrich (v0.6) was used for peak calling using multiple replicates for 1 condition with default threshold. PCR duplicated reads and reads mapping to mitochondrial DNA and the Y chromosome were filtered. Genomic location of peaks was determined using R package ChIPseeker (v1.22.1). The IGV was used to analyze the genomic context of specific differentially accessible regions. Peaks called from ATAC-seq profiles were annotated using ChIPseeker. In detail, the “TxDb.Mmusculus.UCSC.mm10.knowGene” was used as the annotation database, and the promoter region was defined as ± 3 kb around the TSS. Affinity-based analysis was performed using R package Diffbind (v2.14.0) with the DESeq2 method (V1.26.0). For T_conv_ cells, significant differential peaks were defined as having a |log_2_ fold change| > 2 with an adjusted *P* value less than or equal to 0.01 in Lck^Cre^R2D^fl/fl^ CD4^+^ T_conv_ cells compared with those in R2D^fl/fl^ CD4^+^ T_conv_ cells. For Tregs, we used a *P* value less than or equal to 0.01 as a cutoff. TF binding motif analysis of ATAC-seq data was performed using HOMER v4.7.217. Only known motifs from HOMER’s motif database were considered. All ATAC-seq data were deposited in the NCBI’s Gene Expression Omnibus database (GEO accession GSE156927; https://www.ncbi.nlm.nih.gov/geo/query/acc.cgi?acc=GSE156927).

### Statistics.

All statistical analyses were conducted using GraphPad Prism 7 (GraphPad software Inc.). Data were presented as mean ± SD. Statistical differences between 2 populations were calculated by *t* test (2-tailed) including multiple *t* test, unpaired *t* test, or paired *t* test. For multiple populations’ comparison, 2-way ANOVA with Holm-Šidák multiple-comparisons test was used. *P* < 0.05 was considered statistically significant.

### Study approval.

Human samples study (protocol 2006-P-0298) was approved by BIDMC IRB. Informed consent was obtained from all study subjects. All animal procedures were approved by the IACUC of BIDMC, Harvard Medical School. All mice were maintained in an SPF animal facility (BIDMC). All mice were genotyped to validate claimed strain.

## Author contributions

WP designed and performed experiments, analyzed data, and wrote the manuscript. AS helped to perform the human experiments. AF generated the conditional KO mice. YZ analyzed the ATAC-seq data. CB and NY helped to perform flow cytometry staining and cell sorting. MGT analyzed data, provided critical expertise, and edited the manuscript. GCT conceived and supervised the study, interpreted data, and wrote the manuscript.

## Supplementary Material

Supplemental data

Supplemental Table 1

## Figures and Tables

**Table 1:**
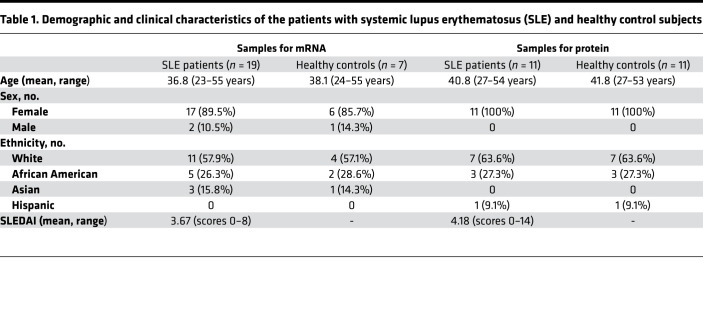
Demographic and clinical characteristics of the patients with systemic lupus erythematosus (SLE) and healthy control subjects

## Version 1 09/08/2020

In-Press Preview

## Version 2 10/02/2020

Electronic publication
