# Supplementary material for: PPP2R2D suppresses IL-2 production and Treg function
Source: JCI Insight. 2020 Oct 2;5(19):e138215. doi: 10.1172/jci.insight.138215 (PMC7566706; doi:10.1172/jci.insight.138215)
Supplement: Supplemental data [file jciinsight-5-138215-s050.pdf]

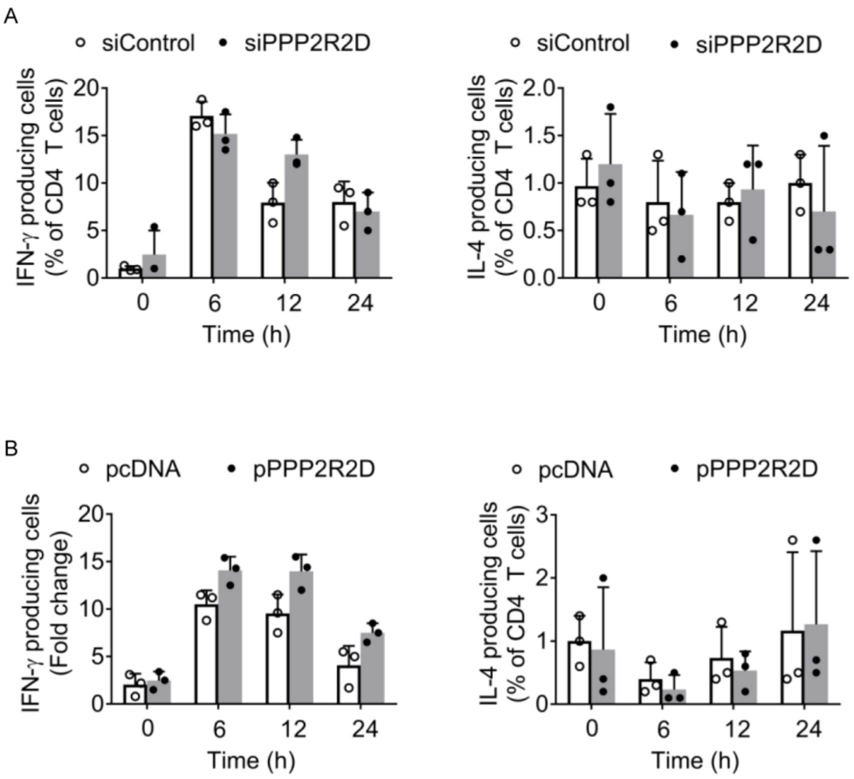

Supplemental Figure 1. PPP2R2D does not affect human IFN-γ and IL-4 producing T cells. Human T cells derived from PBMCs of healthy subjects were subjected to silencing of PPP2R2D (**A**) or to transfecting with PPP2R2D plasmid (**B**), and rested overnight before stimulation with anti-CD3 (OKT3) and anti-CD28 for indicated time. Intracellular staining of IFN-γ and IL-4 production in T cells were analyzed by FACS.

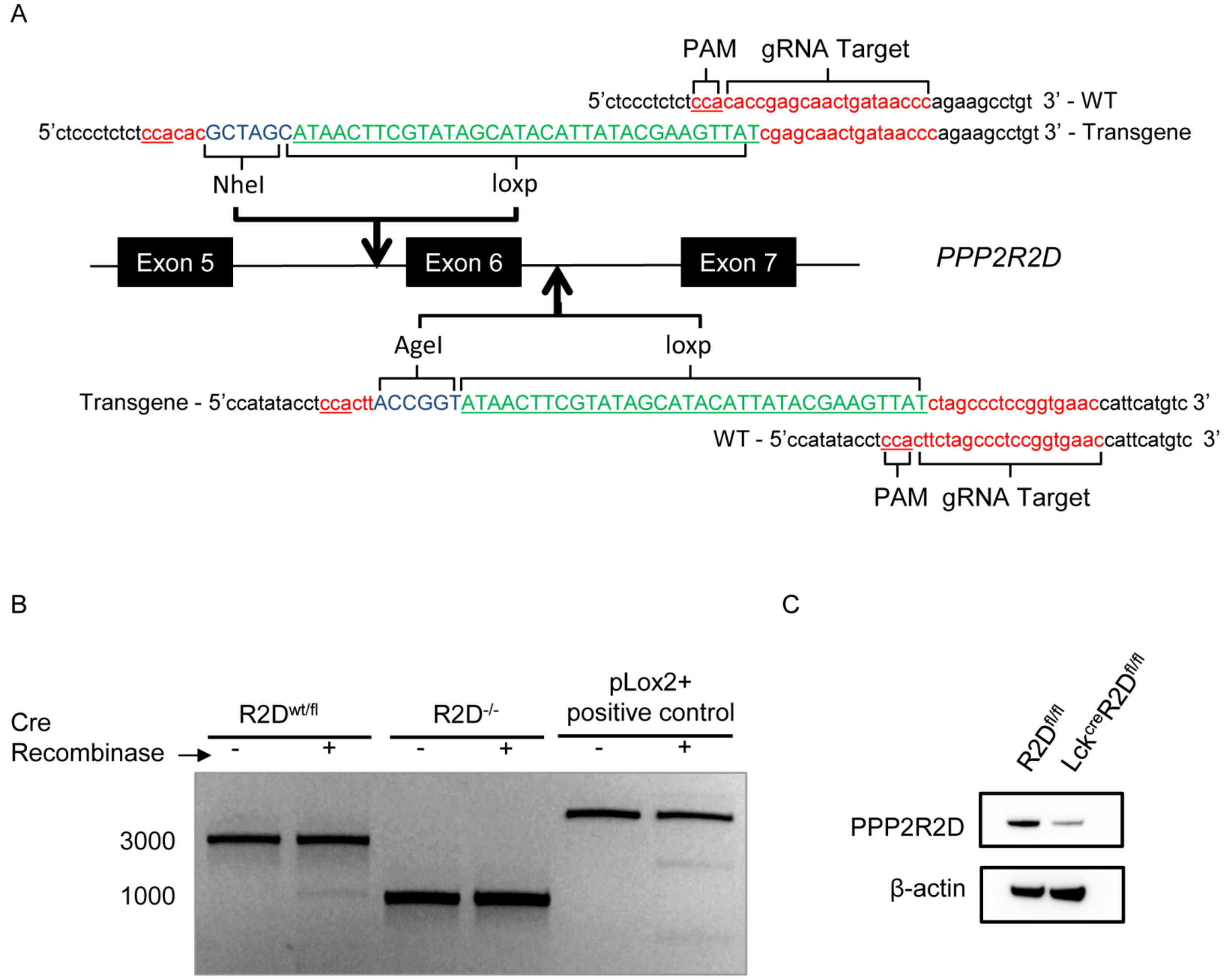

Supplemental Figure 2. Strategy to generate a PPP2R2D a conditional knockout mouse. (**A**) Graphical illustration that gRNAs targeting intronic regions flanking Exon 6 of *PPP2R2D*, and donor single stranded DNA oligonucleotides each containing a loxp consensus sequence and restriction enzyme sites were introduced into pronuclear zygotes in order to generate R2D$^{fl/fl}$ mice. (**B**) Excision of Exon 6 was confirmed by *in vitro* Cre recombination of PCR products amplified from candidate founders. DNA was isolated from R2D$^{wt/fl}$ and R2D$^{-/-}$ mice and amplified by PCR using the primers containing the restriction enzyme sites shown in (**A**). Then the PCR products were incubated at 37 ^{o}C in the presence or absence of Cre recombinase. Linearized pLox2+ DNA which is 3,625 bp in length, with a loxP site approximately 400 bp from each end serves as a positive control. (**C**) Western blot analysis of PPP2R2D expression in T cells which were isolated from R2D$^{fl/fl}$ and Lck^{cre}R2D$^{fl/fl}$ mice.

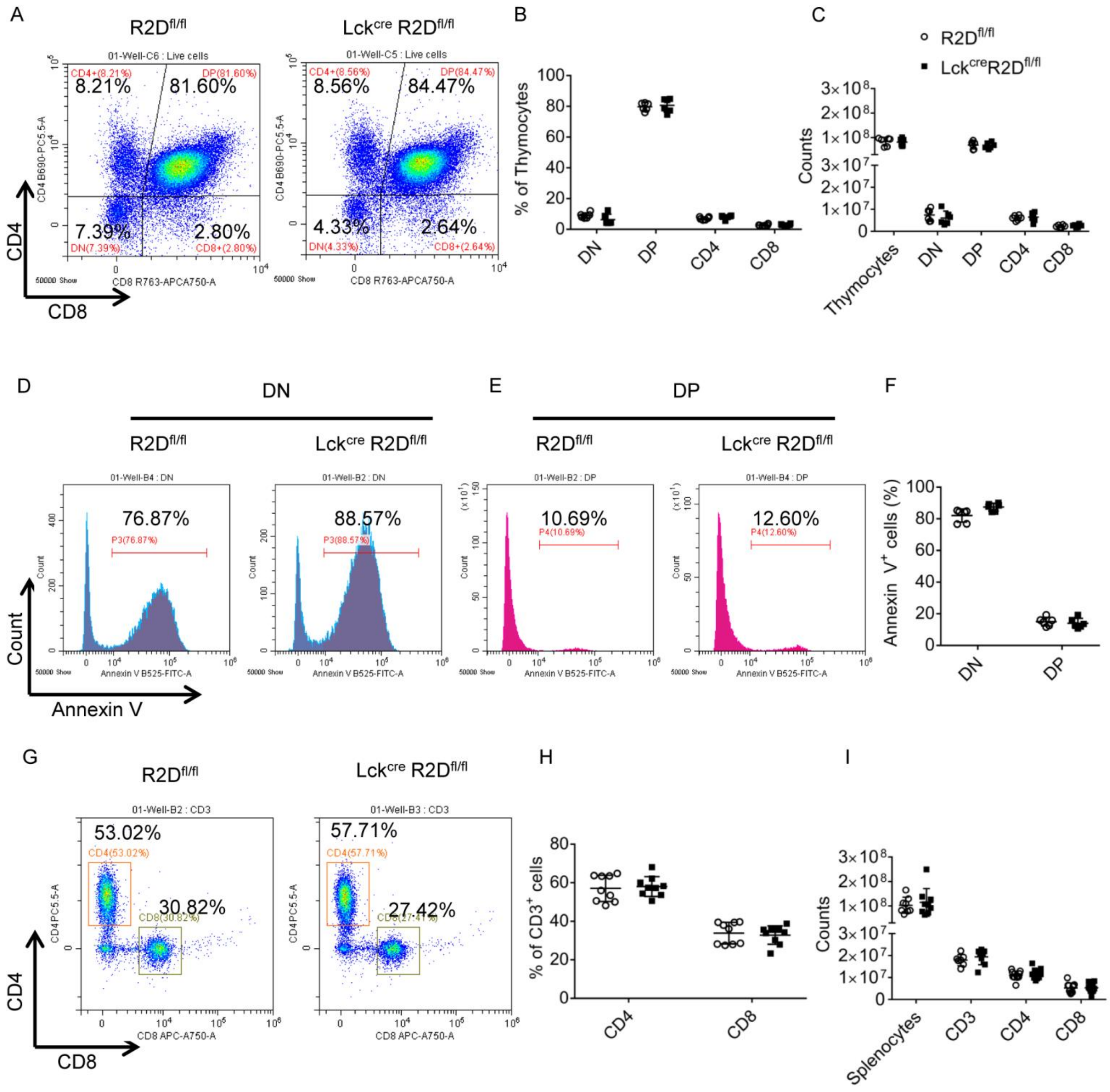

Supplemental Figure 3. PPP2R2D deficiency in T cells does not impair T cell development in the thymus and the subset distribution in the spleen. (**A-C**) Thymocytes isolated from R2D$^{fl/fl}$ or Lck^{cre}R2D$^{fl/fl}$ mice were stained with CD4 and CD8 antibodies and analyzed by flow cytometry. (**A**) Representative flow cytometry plots. Cumulative data (n=6 mice/group) depicting the percentages (**B**) and absolute numbers (**C**) of thymocyte cell populations. DN: Double negative; DP: Double positive. (**D-F**) Thymocytes isolated from R2D$^{fl/fl}$ and Lck^{cre}R2D$^{fl/fl}$ mice were stimulated with CD3 antibody (1 µg/ml) overnight, and subsequently stained with Annexin V, CD4 and CD8 antibodies followed by FACS analysis. (**D and E**) Representative flow cytometry plots. (**F**) Cumulative data (n=6 mice/group) depicting the percentage of Annexin V-positive DN or DP thymocytes. (**G-I**) Splenocytes isolated from R2D$^{fl/fl}$

or $Lck^{cre}R2D^{fl/fl}$ mice were stained with CD3, CD4 and CD8 antibodies and analyzed by flow cytometry. (**G**) Representative flow cytometry plots. Cumulative data (n = 9-11 mice/group) depicting the percentages (**H**) and absolute numbers (**I**) of splenic CD3, CD4 or CD8 positive cells.

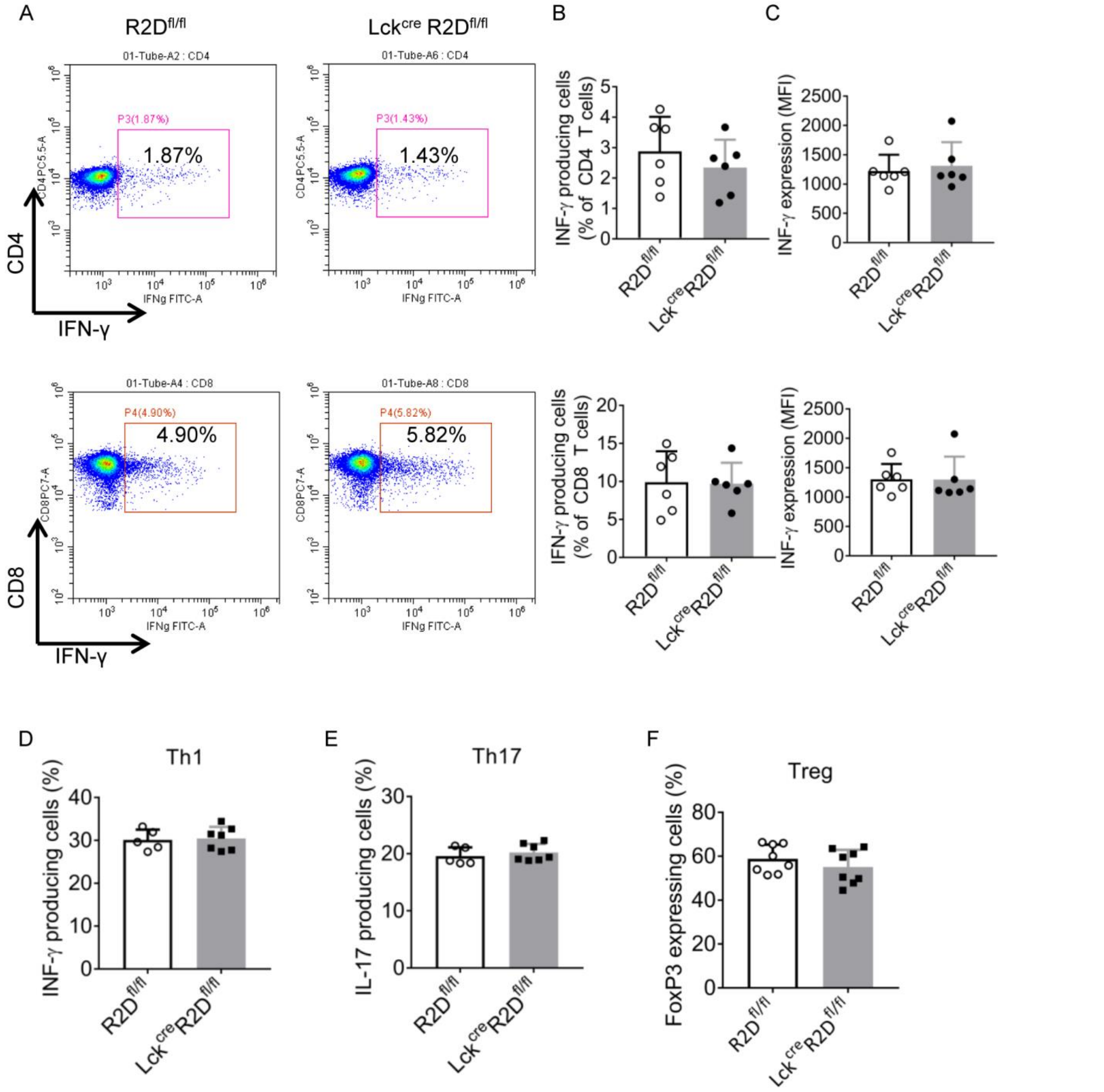

Supplemental Figure 4. PPP2R2D does not affect IFN-γ production, and *in vitro* Th1, Th17 and Treg differentiation in murine T cells. (**A-C**) Splenic CD4 or CD8 T cells were stimulated with phorbol myristate acetate (PMA) /ionomycin, and brefeldin A for 4 hours before subjected to fluorescence-activated cell sorting (FACS) analysis of intracellular staining of IFN-γ production. Representative flow cytometry plots were shown in (**A**). Cumulative data (*n* = 6 mice/group) from individual mice depicting the percentages of IFN-γ -producing cells (**B**) and the expression of IFN-γ (**C**) were presented. MFI: mean fluorescence intensity. (**D-F**) R2D$^{fl/fl}$ and Lck^{cre}R2D$^{fl/fl}$ naive CD4^{+} T cells were cultured under Th1 (**D**)-, Th17 (**E**)- or T$_{reg}$ (**F**) -polarizing conditions for 3 days *in vitro* before FACS analysis.

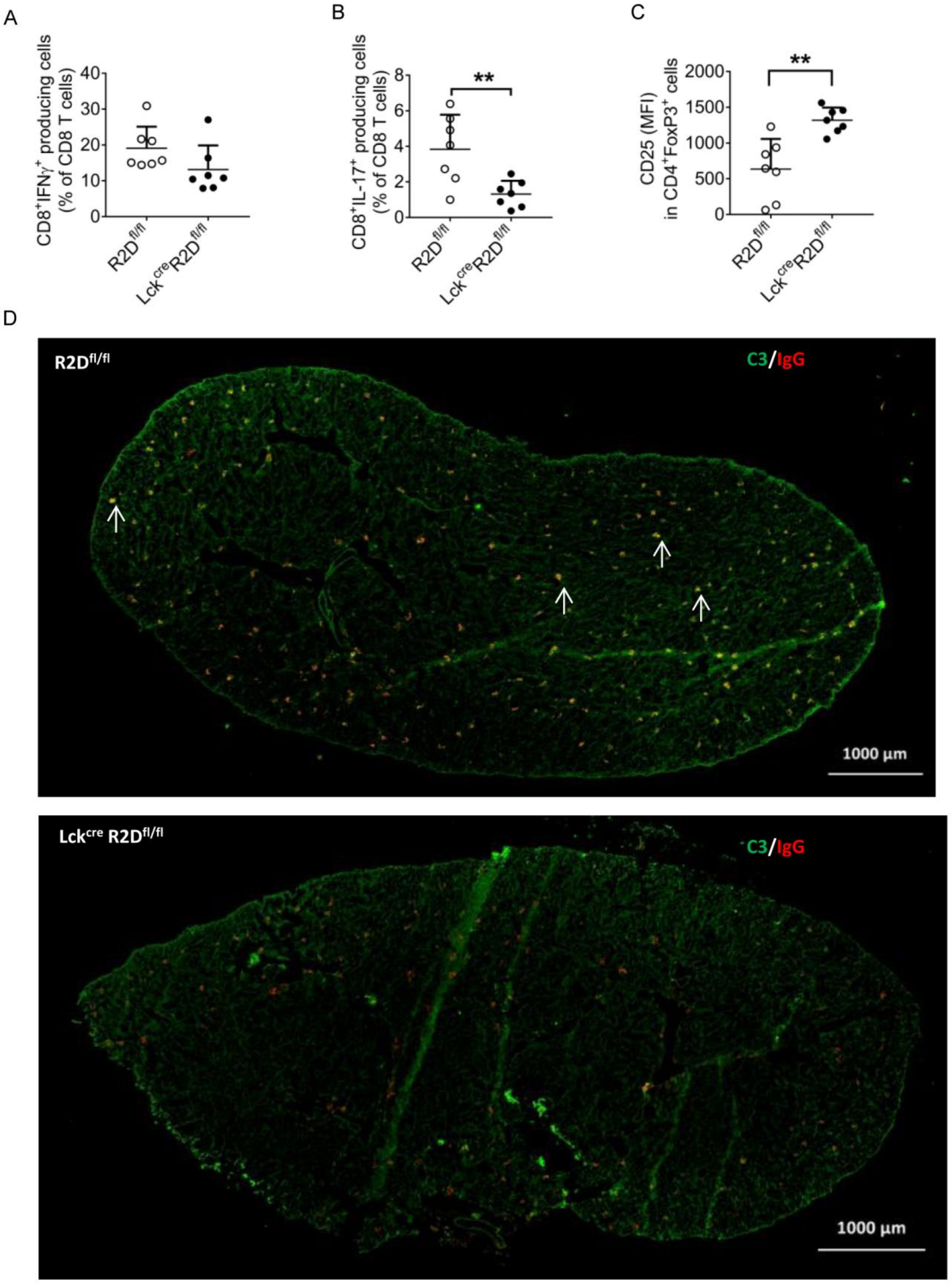

Supplemental Figure 5. Loss of PPP2R2D in T cells decreases imiquimod-induced lupus-like pathology in mice. Topical imiquimod was applied to the skin of ear of R2D$^{fl/fl}$ and Lck^{cre}R2D$^{fl/fl}$ mice (*n* = 7/group) for 8 weeks. FACS analysis of the percentage of CD3^{+}CD8^{+}IFN-γ^{+} (**A**) and CD3^{+}CD8^{+}IL-17A^{+} (**B)** cells in spleens. (**C**) The expression levels of T$_{reg}$ cell markers CD25 in splenic T$_{reg}$ (CD3^{+}CD4^{+}FoxP3^{+}) cells were determined by FACS. (**D**) Representative images of immunofluorescence staining for C3 and IgG from an entire coronal section of kidney. Scale bar: 1000 µm. Arrows point to glomeruli. ***P*<0.01 using Unpaired t-test.

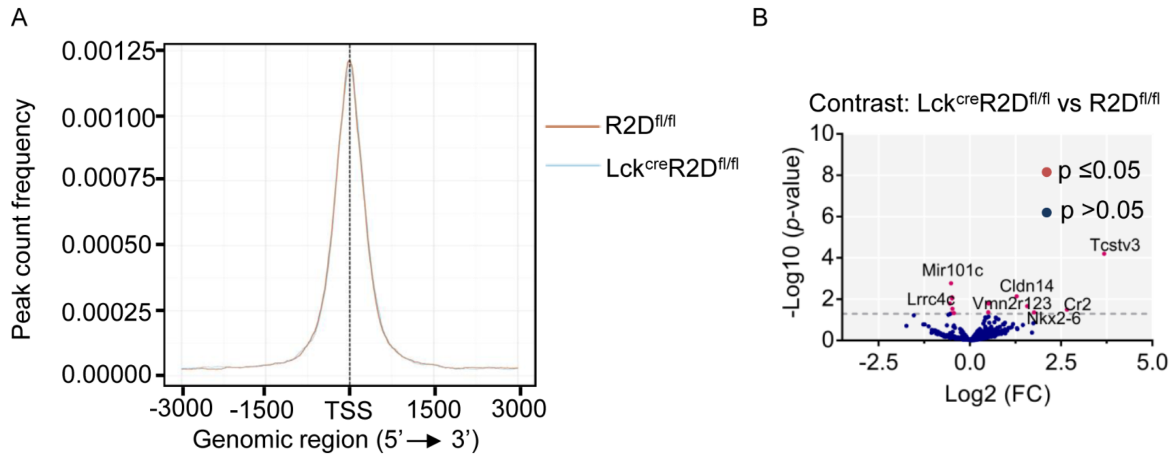

Supplemental Figure 6. Chromatin accessibility profiles of T_{reg} cells with or without PPP2R2D expression using ATAC-seq analysis. CD4 T_{reg} cells were sorted out from spleens of $R2D^{fl/fl}$ or $Lck^{cre}R2D^{fl/fl}$ mice (*n* = 2 mice/group) by flow cytometry, and *ex vivo* stimulated by IL-2 and plate bound CD3 and CD28 antibodies for 4 hours before subjected to ATAC-seq. (**A**) Histogram showing the distance from the nearest transcription start site (TSS) for all ATAC-seq peaks. (**B**) Volcano plot showing differential chromatin accessibility in CD4 T_{reg} cells isolated from $R2D^{fl/fl}$ (wild-type) and $Lck^{cre}R2D^{fl/fl}$ (Knockout) mice. Fold change (FC) is calculated as log2 ($Lck^{cre}R2D^{fl/fl}$/ $R2D^{fl/fl}$). Red dots indicate sites that were significantly different (P value ≤ 0.05).

Supplemental Table 1 The sites of differential accessibility between R2D^{flfl} and Lck^{cre}R2D^{flfl} Tconv cells

(Please see a separate excel file)

Supplemental Table 2 The sequences of primers used for qPCR.

Primer Names	Sequences (5’->3’)
PPP2R2A	Forward: GGTGGTAGAGTTGTCATCTTTCAA<br>Reverse: TCTCCTCTGCTATGAGACTGGA
PPP2R2B	Forward: ATCCTGCCACCATCACAAC<br>Reverse: GCGTTGGCAAATACTCTTCG
PPP2R2C	Forward: AGTTCAACCACACGGGAGAG<br>Reverse: TGGGGCGCATTTTTACTC
PPP2R2D	Forward: TGGCACTTAGAAATCACAGATAGAA<br>Reverse: AACTCGGCTGCAGTGATGA
PPP2R3A	Forward: TTTATGAAATGGGGAAAATTGC<br>Reverse: TGGGGGCTTTCCAATAGAG
PPP2R3B	Forward: GAAGGCTGGACAGCATGG<br>Reverse: CCTGCAGCGTGATCTTCC
PPP2R3C	Forward: ACGAAAACTTTTTGAAGGTTGG<br>Reverse: GACTTTTGCTGTGAAAAATTGCT
PPP2R5A	Forward: CATTGATCAGAAATTCGTACAACA<br>Reverse: CACGTTCTCTGGGATCTTCAC
PPP2R5B	Forward: CGCAAACAGTGCAACCAC<br>Reverse: ACACCATTGAAGTGCTCGAA
PPP2R5C	Forward: CCGTGGTCCTTCTCCATATTC<br>Reverse: GAGGACGCAACACTGACGTA
PPP2R5D	Forward: TGAGTGTCTACCACCCTCAGC<br>Reverse: CTTGGGCCAAAACTTGAGAA
PPP2R5E	Forward: TGGATTCTTCCCCAGAAGC<br>Reverse: TCCACTGATGGAGGAGTAGTTG
IL-2	Forward: GAATCCCAAACTCACCAGGATGCTC<br>Reverse: TAGCACTTCCTCCAGAGGTTTGAG
β-actin	Forward: AGCACTGTGTTGGCGTACAG<br>Reverse: AGAGCTACGAGCTGCCTGAC
Mouse PPP2R2D	Forward: GACGACTTCGATACCCATTTAG<br>Reverse: CGTGGACTTGCTTCTACCATAA
Mouse IL-2	Forward: AGCAGCTGTTGATGGACCTA<br>Reverse: CGCAGAGGTCCAAGTTCAT
Mouse β-actin	Forward: CTAAGGCCAACCGTGAAAAG<br>Reverse: ACCAGAGGCATACAGGGACA

Supplemental Table 3 Antibodies used for FACS.

Antibody-Conjugate	Company (clone)	Dilution
**Figure 1, E and F; Supplemental Figure 1, A and B**		
CD3-PE/Cy7	BioLegend (UCHT1)	1:100
IL-2-APC	BioLegend (MQ1-17H12)	1:50
IFN-γ-Bv421	BioLegend (4S.B3)	1:50
IL-4-PE	BioLegend (8D4-8)	1:50
**Supplemental Figure 3**		
CD3-PE/Cy7	BioLegend (17A2)	1:100
CD4-Percp/Cy5.5	BioLegend (GK1.5)	1:100
CD8-APC/Cy7	BD Pharmingen (53-6.7)	1:100
**Figure 4, A-C; Supplemental Figure 4, A-C**		
CD3-APC/Cy7	BioLegend (17A2)	1:100
CD4-Percp/Cy5.5	BioLegend (GK1.5)	1:100
CD8-PE/Cy7	BioLegend (53-6.7)	1:100
IL-2-APC	BioLegend (JES6-5H4)	1:50
IFN-γ-FITC	BioLegend (XMG1.2)	1:50
**Supplemental Figure 4, D-F**		
CD4-PE/Cy7	BioLegend (RM4-5)	1:100
IFN-γ -Bv421 (Th1)	BioLegend (XMG1.2)	1:50
IL-17A-PE (Th17)	BioLegend (TC11-18H10.1)	1:50
or Foxp3-PE (Treg)	eBioscience (FJK-16s)	1:50
**Figure 5, B and C; Supplemental Figure 5, A and B**		
CD4-FITC	BioLegend (RM4-5)	1:100
CD8-APC eFluo780	eBioscience (SK1)	1:100
Thy1.2-PE/Cy5	BioLegend (53-2.1)	1:100
IL-17A-PE	BioLegend (TC11-18H10.1)	1:50
IFN-γ-Pacific Blue	BioLegend (4S.B3)	1:50
**Figure 5, D and E**		
CD3-PE/Cy7	BioLegend (17A2)	1:100
CD4-PercpeFloor 710	Invitrogen (GK1.5)	1:100
CD8-APC/Cy7	BD Pharmingen (53-6.7)	1:100
FoxP3-AF488	BioLegend (MF-14)	1:50
IL-2-APC	BioLegend (JES6-5H4)	1:50
**Figure 5, F and G; Supplemental Figure 5C**		
CD3-PE/Cy7	BioLegend (17A2)	1:100
CD4-PercpeFloor 710	Invitrogen (SK3)	1:100
CD8-APC/Cy7	BD Pharmingen (53-6.7)	1:100

CD25-FITC	BioLegend (PC61)	1:100
FoxP3-AF488	BioLegend (MF-14)	1:50
CTLA4-Bv421	BioLegend (BNI3)	1:50
GITR-PE	BioLegend (DTA-1)	1:50
**Figure 3; Figure 6, A-D,; Supplemental Figure 6, A and B**		
Thy1.2-PE/Cy7	BioLegend (53-2.1)	1:100
CD4-Percp/Cy5.5	BioLegend (GK1.5)	1:100
CD8-APC/Cy7	BD Pharmingen (53-6.7)	1:100
CD25-FITC	BioLegend (PC61)	1:100
CD127-PE/Dazzle	BioLegend (A7R34)	1:100
**Figure 6, E-H**		
CD4-Percp/Cy5.5	BioLegend (GK1.5)	1:100
CD8-APC/Cy7	BD Pharmingen (53-6.7)	1:100
IFN-γ-PE	BioLegend (XMG1.2)	1:50
